# The metacontrol of event segmentation—A neurophysiological and behavioral perspective

**DOI:** 10.1002/hbm.26727

**Published:** 2024-07-30

**Authors:** Xianzhen Zhou, Foroogh Ghorbani, Veit Roessner, Bernhard Hommel, Astrid Prochnow, Christian Beste

**Affiliations:** ^1^ Cognitive Neurophysiology, Department of Child and Adolescent Psychiatry, Faculty of Medicine, TU Dresden Dresden Germany; ^2^ School of Psychology Shandong Normal University Jinan China

**Keywords:** cognitive control, EEG, event segmentation, fronto‐polar cortex, metacontrol, MVPA

## Abstract

During our everyday life, the constant flow of information is divided into discrete events, a process conceptualized in Event Segmentation Theory (EST). How people perform event segmentation and the resulting granularity of encapsulated segments likely depends on their metacontrol style. Yet, the underlying neural mechanisms remain undetermined. The current study examines how the metacontrol style affects event segmentation through the analysis of EEG data using multivariate pattern analysis (MVPA) and source localization analysis. We instructed two groups of healthy participants to either segment a movie as fine‐grained as possible (fine‐grain group) or provided no such instruction (free‐segmentation group). The fine‐grain group showed more segments and a higher likelihood to set event boundaries upon scene changes, which supports the notion that cognitive control influences segmentation granularity. On a neural level, representational dynamics were decodable 400 ms prior to the decision to close a segment and open a new one, and especially fronto‐polar regions (BA10) were associated with this representational dynamic. Groups differed in their use of this representational dynamics to guide behavior and there was a higher sensitivity to incoming information in the Fine‐grain group. Moreover, a higher likelihood to set event boundaries was reflected by activity increases in the insular cortex suggesting an increased monitoring of potentially relevant upcoming events. The study connects the EST with the metacontrol framework and relates these to overarching neural concepts of prefrontal cortex function.

## Graphical abstract

The study delineates the representational neural dynamics how people are able to vary their mode with which they structure incoming information into encapsulated episodes within the prefrontal cortex.

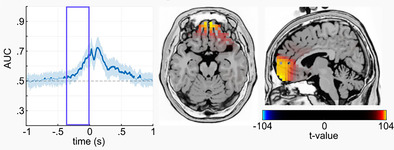

**Practitioner Points**

- The current study connects the established event segmentation theory (EST) with the concept of metacontrol, suggesting that increased cognitive control increases the likelihood to set event boundaries.
- The study delineates the neural processes supporting how people are able to vary their mode with which they structure incoming information into encapsulated episodes to enable goal‐directed behavior.
- The study connects different conceptual frameworks (i.e., event segmentation theory and the metacontrol framework) considering overarching concepts of prefrontal cortex function.

## 1 INTRODUCTION

In everyday life, we are exposed to a constant flow of information, which our brain partitions into distinct events. Event segmentation theory (EST, Zacks et al., 2007) provides a conceptualization of this process based on two mechanistic elements: the working event model, representing the current situation, and event schemata, which contains previously acquired knowledge about typical event progression (Radvansky & Zacks, 2014; Zacks & Sargent, 2010). Working event models are short‐term representations, comparing inputs from the environment with information from event schemata (Richmond & Zacks, 2017; Zacks, 2019). When the obtained input deviates from the expected input, the resulting prediction error leads to the closure of the current event segment, and the opening of a new one, leading to the occurrence of event boundaries (Richmond & Zacks, 2017). As a result, the continuous stream of perception is divided into discrete events by these boundaries (Radvansky & Zacks, 2017) where smaller, detailed events can be combined into more generalized events (Radvansky & Zacks, 2014). For instance, buying a coffee involves entering the cafe, ordering the coffee, and leaving the cafe, each of which can be further subdivided into smaller constituent events.

Interestingly for our purposes, people can be instructed to segment continuous streams into either (many) finer‐grained or (fewer) coarser‐grained segments (Baldassano et al., 2017; Kurby & Zacks, 2011). This hierarchy of events suggests that the segmentation process is under cognitive control. Indeed, it has been suggested that people may adopt various “metacontrol” modes (Beste et al., 2018; Goschke & Bolte, 2014; Hommel, 2015; Hommel & Colzato, 2017a, 2017b) that render information processing either more focused on details or more focused on the broader context. Thus, the currently applied metacontrol mode might influence event segmentation: If the focus is more on details with a high need for top‐down cognitive control, working event models might become more strict and thus even small deviations from the predicted future might result in setting an event boundary and closing the current event segment. On the other hand, if the focus is rather on the broad context, the working event model might be more flexible and thus deviations from the predicted future have to be larger to set an event boundary, resulting in longer, more integrative event segments. Based on previous work of Prochnow et al. (2024), which investigated the temporal structure of the event segmentation process by means of oscillatory activity in EEG data, and previous work showing that segmenting hierarchical events is reflected a cortical hierarchy of neural states (Geerligs et al., 2022), the current study examines the effects of different metacontrol modes on event segmentation and the underlying temporal neural mechanisms. Doing so it provides mechanistic insights into the cognitive processes and their neurophysiological basis underlying event segmentation. This is also of relevance considering the increasing interest in elucidating the neurophysiological underlying cognitive processes operating on different temporal scales (Golesorkhi et al., 2021; Wolff et al., 2022). To this end, we instructed two groups of healthy subjects while recoding EEG signals: The fine‐grain group was asked to meaningfully segment a movie as fine‐grained as possible while watching it. Opposed to this, the free‐segmentation group was instructed to segment the movie according to what they perceive as meaningfully segments of the same movie. We hypothesize that this metacontrol mode manipulation affects the event segmentation process. More specifically we hypothesize that this manipulation alters how event schemata are employed to construct the working event model. We assumed that the fine‐grain group applies a stricter approach while the free‐segmentation group is expect to apply more flexible/lenient mode, resulting in distinct usage of event schemata. On behavioral level, the fine‐grain group is expected to be more sensitive to situational changes throughout the movie compared to the free‐segmentation group. The fine‐grain group is therefore expected to set more event boundaries.

On neural level, we anticipate that either the temporal neural representation or the brain regions involved in these processes differ between the two groups, or both. To capture the differences between the groups on the neural level, we analyse EEG recordings using multivariate pattern analysis (MVPA) and combine this with source localization methods. A method that accurately distinguishes conditions over time (King & Dehaene, 2014; Takacs et al., 2020; Yu et al., 2023)—such as MVPA—stands the greatest chance of capturing these representational dynamics, but not using simpler EEG analysis methods (e.g., event‐related potentials, etc.). Besides, we want to examine whether neural representations of event models active before the closing of one event segment are completely discarded or affect processes in the subsequently opened event segment. This is relevant, because (i) metacontrol likely affects how well different representations can be shielded from each other to all goal‐directed acting (Goschke & Bolte, 2014; Zhang et al., 2023) and (ii) EST currently does not consider a leakage of information between adjacent events. Importantly, also other theories (Hommel, 2004; Wahlheim & Zacks, 2019) suggest that a current event representation can be affected by preceding event representations if they share some characteristics. Through incorporating another MVPA named temporal generalization, which enables training and testing of datasets at different time points (King & Dehaene, 2014; Petruo et al., 2021), we can elucidate how well event schemata representational dynamics affects upcoming processing of event segments or whether previous event schemata leak into and affect processing of events.

On the neuroanatomical level, we expected that activity modulations in fronto‐polar regions are associated with the described modulations in the representational dynamics. The fronto‐polar cortex has been implicated in the arbitration of exploitation and exploration (Koechlin & Summerfield, 2007; Mansouri et al., 2017), based on a system performing on online monitoring of the relevance of behavioral options. Through directed exploration, it is possible to simultaneously monitor and evaluate multiple hypotheses/scenarios of possibly upcoming behavioral options (Mansouri et al., 2017). This is at the core of event segmentation which is performed to structure environmental information to ultimately enable goal‐directed actions. According to EST, individuals continually compare ongoing perception with predictions, setting boundaries whenever prediction errors arise. In other words, the working event model constantly creates scenarios about the preceding of current situation, which are tested against the perceptual input from the environment. If such a working event model is challenged too much, that is, the prediction turns out wrong, the working event model is updated. Moreover, exploration and exploitation, as described as functions of the fronto‐polar cortex, can also be linked to the concepts of cognitive flexibility and cognitive persistence as described in the metacontrol concept, respectively. Cognitive flexibility involves adapting to change and seeking new options, akin to exploration. Conversely, cognitive persistence entails sticking to a strategy or information, akin to exploitation. Therefore, we hypothesize that fronto‐polar regions are associated with metacontrol‐related modulations of event segmentation processes.

## 2 MATERIALS AND METHODS

### 2.1 Participants

Healthy adults with age ranging from 18 to 30 years were invited to participate in the current study. None of them reported current or past neurological or psychiatric illness, substance abuse or dependence, or current chronic or acute medication. All subjects had normal or corrected‐normal vision. Subjects were divided into two groups which received different instructions for the task to be performed (see Section 2.2). This resulted in a Free‐segmentation group and a Fine‐grain group. All participants were recruited using the databases of University Clinic Carl Gustav Carus and the TU Dresden or advertises. Before testing, all subjects read and signed the informed consent. This study was approved by the local ethics committee in Medical Faculty of the TU Dresden. The behavioral and EEG raw data as well as the code for the data analysis are deposited at https://osf.io/78xyk/?view_only=6d155924939f437c86a376f805e51256.

The Free‐segmentation group consisted of *N* = 45 participants, of which *N* = 6 participants had to be excluded due to issues with the EEG data recording, data quality or being an outlier with respect to the number of responses. In the end, there were *N* = 39 participants included in the data analyses (19 females; mean age 25.67 ± 2.85 years). The Fine‐grain group also consisted of *N* = 45 participants, of which *N* = 3 participants had to be excluded as their behavioral data were outliers regarding the number of responses. Finally, there were *N* = 42 participants in this group used for the data analysis (19 females; mean age 25.17 ± 2.59 years).

### 2.2 Task

Both groups performed a validated event segmentation task (Magliano & Zacks, 2011) with different instructions to modulate the amount of cognitive control exerted in this task. All subjects were shown a movie and instructed to press the space key whenever they found “some meaningful units (e.g., actions, interactions, and goals) were finished and some other meaningful units were about to start”. The difference of instructions between the groups was that the Fine‐grain group was asked to “define these meaningful units as small as possible”, whereas the Free‐segmentation group received no specific instructions regarding the extent of the units. First, both groups performed a supervised exercise, using different videos for each group: for the Free‐segmentation group, it was a man assembling ‘Duplo’ construction blocks (Zacks et al., 2009); for the Fine‐grain group, the practice video presented a woman preparing breakfast in the kitchen (Bailey, Kurby, et al., 2013; Bailey, Zacks, et al., 2013). The training video watched by the Fine‐grain group contains more action, making it comparatively easier for participants to segment it into smaller events. Afterward, all participants performed the task with the group‐specific instruction on the short movie ‘The Red Balloon’ (Anon, 1956) and the EEG was recorded during the task. This film is well‐suited for event segmentation analysis due to its limited use of spoken language, frequent changes in the situation, and minimal temporal jumps (Magliano & Zacks, 2011; Zacks et al., 2009, 2010). The movie was separated into four episodes (lengths: 463.3, 468.4, 446.2, and 600.6 s) with breaks between them to offer relax time for participants. ‘Presentation’ software (NeuroBehavioral Systems Inc.) was used to present all videos and to record the participants' responses. Thus, the entire experimental implementation was similar to a previously published study by Prochnow et al. (2024).

### 2.3 Predictors of event segmentation—situational changes

Nine types of situational changes in ‘The Red Balloon’ movie had been specified and scored frame by frame by previous work (Zacks et al., 2009) and were also used in the current data analysis. Moreover, this situational change coding has also been applied in subsequent studies (Kurby et al., 2014; Prochnow et al., 2024; Zacks et al., 2010). Situational changes were characterized as follows: (i) “Temporal changes” were characterized as instances where the frame immediately following a cut was disconnected from the frame preceding the cut in terms of time. “Spatial changes” were categorized into two types: (ii) “Large space changes” denoted situations where the character's position between two consecutive frames had significantly shifted, and (iii) “Small space changes” referred to any alterations in the camera's perspective or location. (iv) “Character changes” were noted when the primary focus of an action or behavior in a scene shifted to a different character or an animated character compared to the preceding frame. (v) “Character‐character changes” represented instances when character's interaction with each other changed, such as moving closer, engaging in conversation, making gestures, or having physical contact. (vi) “Character‐object changes” were recorded when there was a change in the dynamics between the character and objects, or when the character started using the object in a different manner compared to the previous frame. (vii) “Cause changes” were identified when the actions depicted in the current frame were not a direct result of events shown in the previous frame. (viii) “Goal changes” were coded when a character's behavior associated with a specific goal differed from that portrayed in the preceding frame. (ix) “Scene changes” encompassed moments where an entirely new shot replaced the previous one. For behavioral data analysis, following previous studies (Zacks et al., 2009, 2010), all movie clips were divided into 2 s intervals (982 intervals in total). Each interval was either categorized as boundary interval (BI) or no‐boundary interval (NBI), depending on whether there was a button press during the interval or not. Further, the number of situational changes during each interval was also counted, both separately and collectively by situational change type.

### 2.4 Analysis of behavioral performance in the task

To analyze the behavioral data statistically, mixed‐effects logistic regressions (R version 4.2.1, ‘glmer’ function) were conducted to assess the impact of situational changes on segmentation patterns. Following previous work (Zacks et al., 2009), two mixed‐effects regression models were run: one to predict segmentation probability based on the number of changes, and the other to examine the relationship between each type of change and segmentation pattern. Both models included a random effect for subjects to accommodate inter‐subject variability, and odds ratios were computed from fixed effect coefficients to compare predictor influences. For the first model, groups and the total number of situational changes (ranging from 0 to 5) within each 2 s interval served as the predictors, with the outcome being participant responses indicating event boundaries or no response. For the second model, predictors were groups and binary indicators (1 or 0) for the presence or absence of each of the nine types of situational changes within a 2 s interval, with the same outcome variable as in the first model. Additionally, the variance inflation factor (VIF) was calculated to assess multicollinearity among predictors.

### 2.5 EEG recording and pre‐processing

While participants were watching the movie, elastic caps (EasyCap Inc.) with 60 Ag/AgCl electrodes were used to record EEG signals (reference electrode at Fpz, ground electrode at *θ* = 58, *ϕ* = 78). BrainAmp amplifiers (Brain Products Inc.) were used to amplify EEG signals and the electrode impedances were kept under 5 kΩ. The online sampling rate was 500 Hz, which was down‐sampled to 300 Hz during offline pre‐processing. Data pre‐processing was conducted by the “Automagic” pipeline (Pedroni et al., 2019) and EEGLAB (Delorme & Makeig, 2004) running on Matlab 2019a (The MathWorks Corp.). First, flat channels were detected and removed, and then an average referencing was applied to EEG data. Next, the PREP preprocessing pipeline and the EEGLAB ‘clean_rawdata()’ pipeline were applied. Line noise at 50 Hz was removed and then a robust average reference was applied after removing contamination by bad channels. A finite impulse response (FIR) high‐pass filter (0.5 Hz, order 1286, stop‐band attenuation −80 dB, transition band 0.25–0.75 Hz) was applied to identify and remove channels that were flat‐lined, noisy, or outliers. A lowpass filter of 40 Hz (sinc FIR filter; order: 86; Widmann et al., 2015) was applied to remove electromyographic (EMG) artifacts. Electro‐oculographic (EOG) artifacts were removed using a subtraction method (Parra et al., 2005). Muscle, cardiac, and remaining ocular artifacts were categorized and eliminated by Independent Component Analysis (ICA) which was based on Multiple Artifact Rejection Algorithm (MARA; Winkler et al., 2011, 2014). Artifact Subspace Reconstruction (ASR; burst criterion: 15; Mullen et al., 2013) was used to reconstruct epochs in the segmented data (see below) with abnormally strong power (>15 standard deviations relative to calibration data). Time windows that could not be reconstructed were discarded. In the end, all missing and eliminated channels were interpolated by a spherical method.

**FIGURE 1:**
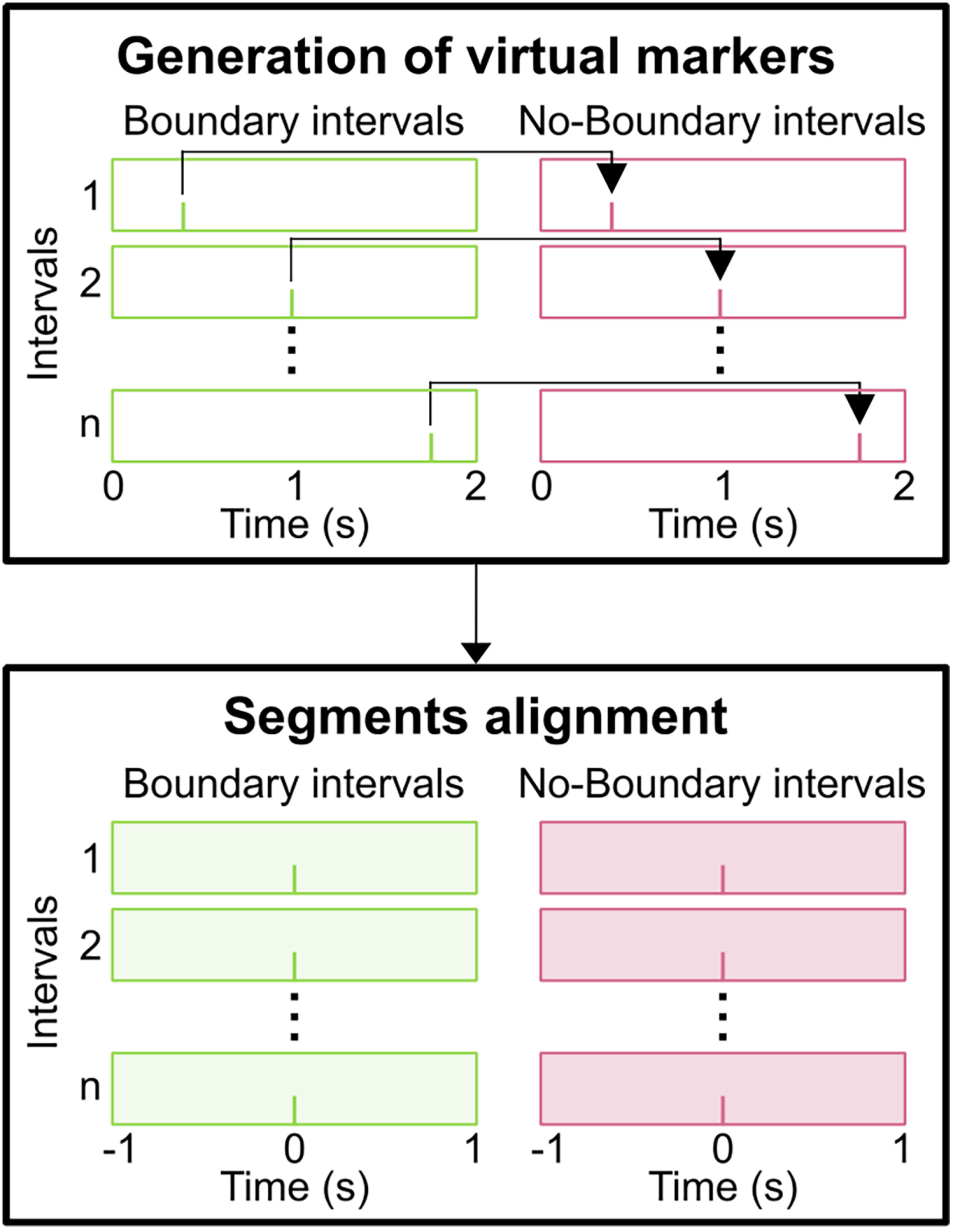
Schematic illustration of the segmentation of neurophysiological data. Boundary intervals are indicated by green color. No‐boundary intervals are indicated by pink color. The markers displayed within these intervals represent the response and virtual markers, respectively. In a first step, boundary intervals were randomly allocated to no‐boundary intervals. Virtual markers were then positioned within the no‐boundary intervals at the same time point within the interval at which the key press occurred in the corresponding boundary interval (upper section). In the next step, data were re‐segmented according to these markers, enabling the analysis of data ranging from −1 to 1 s relative to the marker's position (lower section).

FieldTrip (Oostenveld et al., 2011) was used to conduct the subsequent analysis steps. In order to compare the difference between time windows with indicated event boundaries and without indicated event boundaries, we defined Boundary intervals (BI; with button presses referring to event boundaries) and No‐Boundary intervals (NBI; without button presses referring to no event boundaries), as established in the study by Prochnow et al. (2024). While BI in this way contained response markers (button presses), there were no markers for NBI. Therefore, we constructed virtual markers based on response markers by applying the following steps: (i) In line with behavioral data, we segmented continuous data into 2 s intervals. In all participants, there were more intervals without response markers than intervals with response markers. (ii) For each participant, intervals without response markers were randomly selected in a number equal to the number of intervals with response markers. Intervals with and without response markers were randomly assigned to each other. (iii) The time point of a response marker within the interval was projected as a virtual marker onto the corresponding interval without a response marker assigned in step (ii). This results in corresponding response markers for BI and virtual markers for NBI, the numbers of which is the same in each participant. This study relies on response‐locked data analysis for two key reasons: First, there are no clearly separable stimuli as in standard EEG paradigms, and second, the critical aspect of event segmentation is marked by the timing of motor responses or button presses. Data from −1 to 1 s relative to both types of markers were included in the next analysis steps. A schematic illustration of the segmentation steps is shown in Figure 1.

### 2.6 Classification (MVPA)

To differentiate between BI and NBI across time, we conducted MVPA on time domain data by using the MVPA‐Light toolbox (Treder, 2020). Two distinct analyses were performed for each individual subject, where only signals within the −1 to 1 s relative to button press were fed into the MVPA for each trial: First, a binary classification across time, in which training and testing of the classifier is conducted with data from the same time point, was carried out for each group to pinpoint specific time points that exhibited distinct spatial patterns on the electrode level between BI and NBI. Second, to gain a deeper understanding of the temporal dynamics of the representational content, a temporal generalization MVPA was conducted. The classifier in temporal generalization MVPA is trained on data from one time point *t* and tested on data from the same time point *t* and additionally from every other time point *t*′. This process helps determine if the neural representation identified at time point *t* recurs at time *t*′. The classifier employed in this study was Regularized Linear Discriminant Analysis (LDA, Renton et al., 2022), which was evaluated using a 10‐fold cross‐validation approach with 10 times repeat. As for the other parameters, they were set to the default values in the MVPA‐Light toolbox. The evaluation of classification accuracy was conducted using the area under the curve (AUC), which is a non‐parametric measure of effect size derived from signal detection theory. The AUC value in MVPA indicates the distinction between boundary intervals (BI) and no‐boundary intervals (NBI), with a higher AUC indicating a greater disparity. To identify time points with significant classification performance indicated by the AUC, cluster‐based permutation testing was conducted. This analysis involved 1000 random draws and utilized non‐parametric Wilcoxon tests with a significance level of *p* = .05. The cluster‐level statistic was determined by summing all the Wilcoxon test values within the specified time range. The null value for the AUC was set at a chance level of 0.5, which corresponds to 50%.

### 2.7 Correlational analysis

To examine the connection between behavioral data and neurophysiological data, we employed Pearson correlation analysis. The behavioral data consisted of logistic regression coefficients for each individual, indicating sensitivity to the number of situational changes during the movie. On the other hand, the neurophysiological data comprised the AUC values obtained from the MVPA across time for each subject. Correlation analyses between behavioral coefficients and AUC values reveal the extent to which sensitivity to environmental changes during event segmentation, as indicated by logistic regression coefficients, aligns with neural modulations, as indicated by the distinguishability of NBI and BI in MVPA.

### 2.8 Source localization analysis (sLORETA)

To determine the functional neuroanatomical structures linked to time domain data in the MVPA‐detected time windows, standardized low‐resolution brain electromagnetic tomography (sLORETA (Pascual‐Marqui et al., 2002)) was employed. For each group, we aim to investigate the distinct brain regions that contribute to BI and NBI separately, with a specific focus on examining the time periods with significant classification performance across time before and after a response or a virtual marker, respectively. sLORETA employs a realistic MNI152 head model and divides the intracerebral volume into 6239 voxels with a spatial resolution of 5 mm (Mazziotta et al., 2001). Subsequently, a standardized current density is calculated for each voxel (Fuchs et al., 2002). sLORETA offers a linear solution to the inverse problem without introducing any localization bias (Sekihara et al., 2005). To achieve this, a built‐in voxel‐wise randomization test with 2000 permutations, based on statistical non‐parametric mapping (SnPM), was conducted by sLORETA. The results section displays the voxels located in the MNI brain template that exhibit a significant difference (*p* < .05) between BI and NBI as determined by the analysis.

## 3 RESULTS

### 3.1 Behavioral results

Within each 2‐s interval throughout the entire movie, the number of situational changes was calculated, which is the same for the free‐segmentation and fine‐grain groups. In total, there were 518 intervals where no changes occurred, 278 intervals with a single change, 106 intervals with two changes, 52 intervals with three changes, 29 intervals with four changes, and four intervals with five situational changes.

**FIGURE 2:**
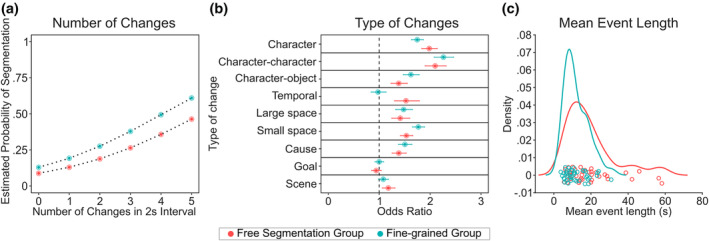
Results of the mixed‐effects logistic regression and mean event length analyses. Data of the free‐segmentation group are represented in orange, data of the fine‐grained group are represented in green. Figure part (a) shows the predicted likelihood of segmentation (*y*‐axis) as a function of the number of situational changes within an interval (*x*‐axis) for both groups. Figure part (b) shows the odds ratios (represented by dots) alongside their corresponding 95% confidence intervals (shown as error bars) for each type of change for both groups. The impact of a factor on segmentation probability is deemed significant when the confidence interval does not encompass 1 (dashed line). Figure part (c) shows the density of average event durations for both groups.

The mixed‐effect logistic regression analysis with a significant intercept (−1.908, *p* < .001) demonstrated significant coefficients for the number of changes (0.471, *p* < .001; OR = 1.602, 95% CI = 1.568–1.637), group (−0.431, *p* < .01; OR = 0.650, 95% CI = 0.483–0.874), and, most importantly, their interaction (−0.032, *p* < .05; OR = 0.968, 95% CI = 0.937–0.999). These findings, as depicted in Figure 2, indicate that the likelihood of segmentation increased with an increasing number of situational changes for both subject groups. However, the rate of this increase was smaller for the free‐segmentation group (intercept: −2.340, *p* < .001, coefficient: 0.439, *p* < .001) compared to the fine‐grain group (intercept: −1.908, *p* < .001, coefficient: 0.471, *p* < .001).

Next, we conducted a second mixed‐effect logistic regression to assess the association between each of the nine types of situational change and event segmentation probability in both groups. In this analysis, there were no concerns regarding multicollinearity as all VIFs remained below 5 (VIF ≤ 4.614). The odds ratios (ORs) for all nine types of situational changes and their interactions with the predictor group are presented in Table 1. The analysis revealed significant interactions between situational change type and group for character, character‐object, small‐space, and temporal changes. In the free‐segmentation group, character changes had a stronger predictive effect on segmentation compared to the fine‐grain group (free: OR = 1.982, 95% CI = 1.833–2.144; fine‐grain: OR = 1.746, 95% CI = 1.631–1.870). Additionally, temporal changes were predictive of segmentation in the free‐segmentation group (OR = 1.526, 95% CI = 1.300–1.790), but not in the fine‐grain group (OR = 0.976, 95% CI = 0.838–1.137). On the other hand, character‐object changes were less predictive of segmentation in the free‐segmentation group (OR = 1.383, 95% CI = 1.231–1.553) compared to the fine‐grain group (OR = 1.621, 95% CI = 1.470–1.787). The same trend was observed for small‐space changes (free: OR = 1.533, 95% CI = 1.414–1.662; fine‐grain: OR = 1.768, 95% CI = 1.651–1.894).

**TABLE 1:** The odds ratio (OR) for type and the interaction between type and group.

Situational change type	OR type (95% CI)	OR type × group (95% CI)
Character	**1.752** (1.636–1.876)	**1.127** (1.016–1.250)
Character–character	**2.268** (2.083–2.470)	0.922 (0.809–1.050)
Character–object	**1.626** (1.475–1.793)	**0.847** (0.728–0.985)
Small‐space	**1.774** (1.656–1.900)	**0.861** (0.774–0.957)
Large‐space	**1.480** (1.323–1.655)	0.954 (0.806–1.130)
Temporal	0.976 (0.838–1.137)	**1.562** (1.252–1.948)
Cause	**1.506** (1.378–1.646)	0.919 (0.803–1.051)
Goal	0.999 (0.915–1.090)	0.942 (0.823–1.077)
Scene	1.082 (0.986–1.188)	1.085 (0.942–1.249)

*Note*: The 95% confidence interval (CI) is given in brackets. Significant ORs are displayed in bold value.

Furthermore, we analyzed the distributions of the likelihood that participants from different groups identified boundaries during both free and fine segmentation, shedding light on the segmentation tendencies exhibited by these distinct groups when processing continuous event sequences. As depicted in Figure 2c, the average duration for the free‐segmentation group was 19.134 s, whereas it was 12.015 s for the fine‐grain group. An independent samples *t*‐test revealed a significant difference between the two groups (*t* = 3.126, *p* = .003, SD = 10.240, df = 79).

### 3.2 Neurophysiological data

**FIGURE 3:**
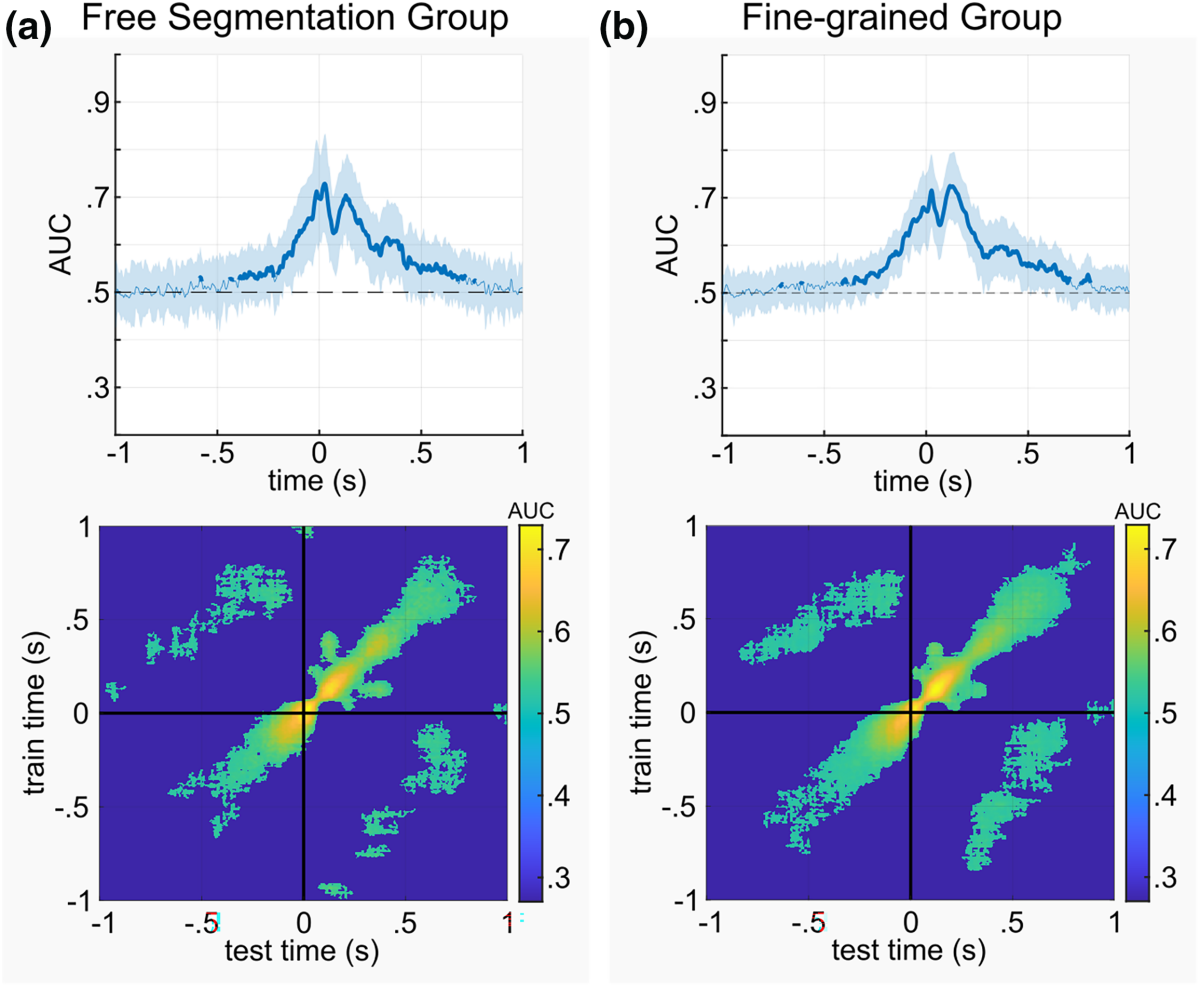
Outcome of MVPA (classes boundary and no‐boundary intervals) for both the free‐segmentation group (a) and the fine‐grained group (b). In the upper panel, the area under the curve (AUC) is depicted for each group, with bold lines denoting statistically significant classifications above chance level. The shading surrounding the lines indicates the AUC's standard deviation across the sample. The lower panel displays the temporal generalization matrices for both groups, with color gradation representing the extent of classification accuracy, as indicated by the AUC.

Figure 3a displays the binary classification performance across time as well as the temporal generalization matrix for the free‐segmentation group, Figure 3b shows this information for the fine‐grain group.

In the free‐segmentation group, the MVPA analysis revealed a significant time window ranging from approximately 400 ms before the event boundary to 680 ms after the event boundary, with an average AUC of .591 (AUC_min_ = .520, AUC_max_ = .728). Regarding the temporal generalization MVPA, about 14% of the classifications resulted in a significant AUC value, with an average AUC of .547 (AUC_min_ = .501, AUC_max_ = .729) for the significant classifications. The temporal generalization analysis revealed that approximately 400 ms before the event boundary off‐diagonal activity was evident for about 250 ms. This degree of off‐diagonal activity was evident until approximately 50 ms before the event boundary. Shortly after the event boundary was set, off‐diagonal activity was lowest before re‐instating approximately 250 ms after the event boundary was set. The average duration of the off‐diagonal activity along the diagonal axis was about 74 ms. An important aspect revealed by the off‐diagonal activity is shown in the upper‐left and the lower‐right quadrant of the temporal generalization matrix (Figure 3a, lower panel). As can be seen, significant decoding was also evident in these quadrants.

In the Fine‐grain group, the significant time window for classification across time was observed from −350 to 707 ms around segmentation with an average AUC of .590 (AUC_min_ = .514, AUC_max_ = .725). Regarding the temporal generalization MVPA, approximately 17% of the classifications showed a significant AUC value, with an average AUC of .541 (AUC_min_ = .505, AUC_max_ = .726) for those significant classifications. The temporal generalization analysis revealed the same pattern of off‐diagonal activity as found in the free‐segmentation group. The average duration of the off‐diagonal activity along the diagonal axis was about 85 ms.

**FIGURE 4:**
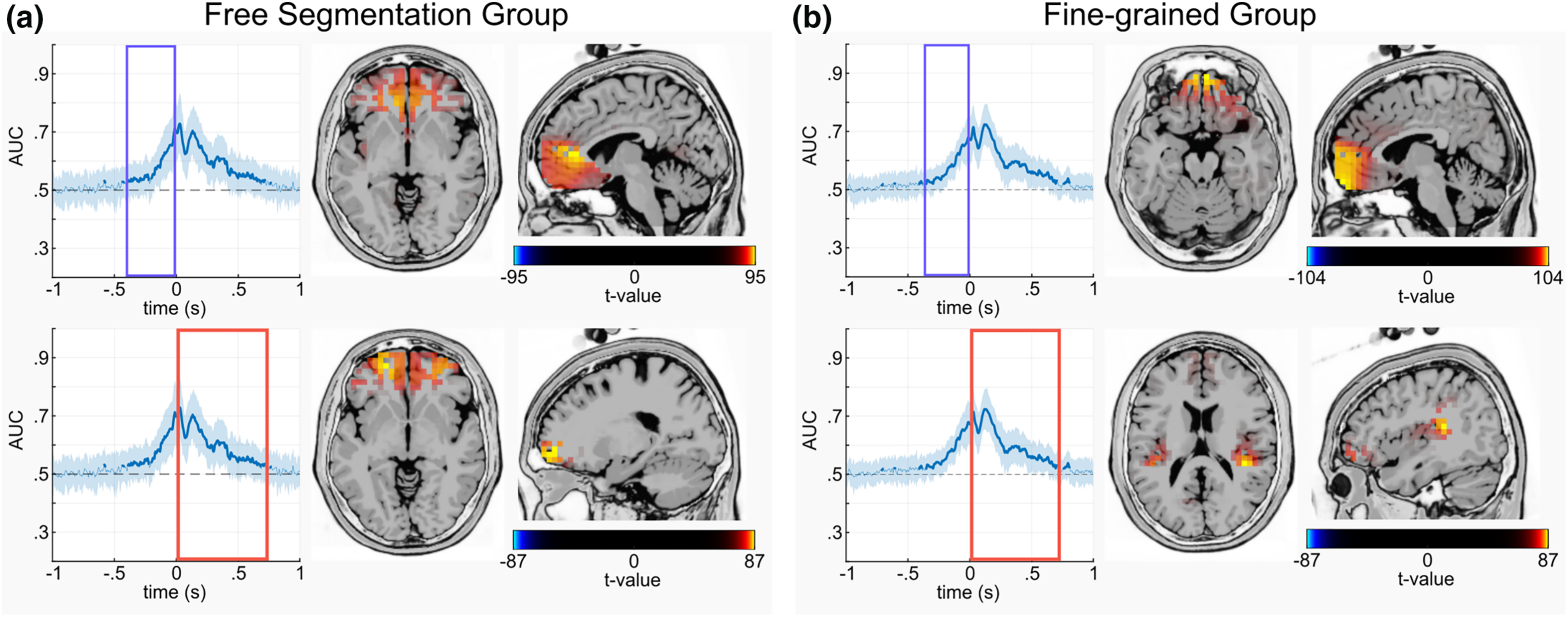
sLORETA outcomes within the time window of significant AUC in MVPA are presented for both the free‐segmentation group (a) and the fine‐grained group (b). In the upper panel, the sLORETA results before the event segmentation are displayed (within blue rectangles time window), while the lower panel shows the sLORETA results after the event segmentation (within red rectangles time window).

Source localization analyses were run for the time period identified to differ significantly between BI and NBI. This was done for the free‐segmentation group and fine‐grain group. During the time window before an event boundary was set, both the free‐segmentation and fine‐grain groups exhibited higher neural activation in BI compared to NBI in the fronto‐polar part of the medial frontal gyrus (BA10) (Figure 4). For the free‐segmentation group, the source localization analysis showed continued higher activity in the fronto‐polar medial frontal gyrus (BA10) for BI compared to NBI during the significant time window after segmentation. For the Fine‐grain group, the source localization analysis indicated higher also activity in the insula (BA13) for BI compared to NBI after the time point of segmentation.

**FIGURE 5:**
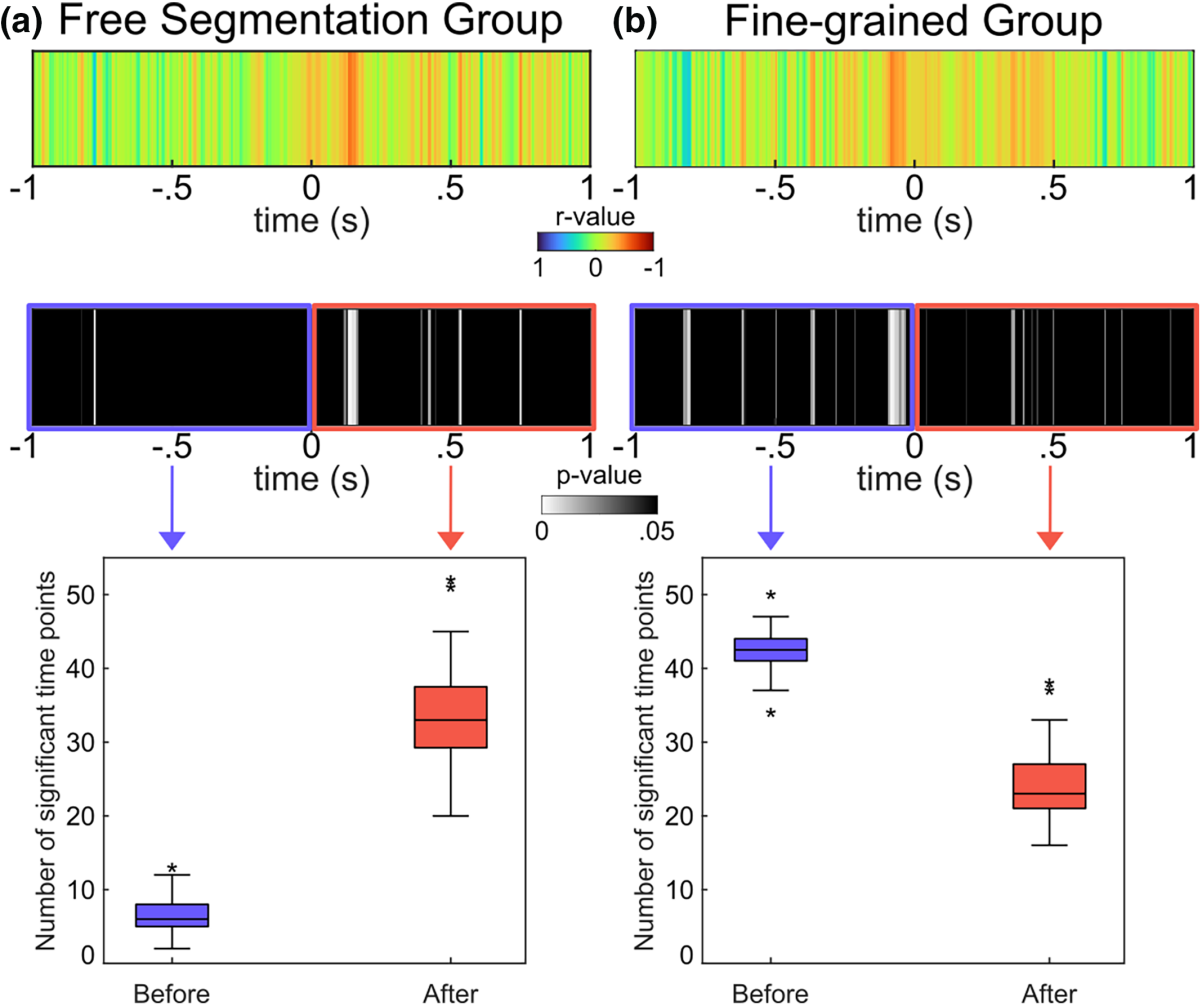
The correlation findings between the slope of logistic regression and the area under the curve (AUC) in MVPA are presented. Figures (a) and (b) provide distinct outcomes for the free‐segmentation and fine‐grained groups. In the upper panel, the outcomes for the correlation coefficient (*r*‐value) are depicted, while the middle panel shows the *p*‐values. The number of significant time points was counted before (blue) and after (red) event segmentation (time point zero) for each individual. The distribution of the number of significant time points for each group and time window is displayed in the lower panel.

To examine the inter‐relation of neural processes and behavioral performance more closely, we performed Pearson correlations using the AUC data and the slope of mixed‐effect logistic regression function delineating the inter‐relation of the degree of situational change and the likelihood to set an event boundary. The results are shown in Figure 5.

As depicted in Figure 5, both the free‐segmentation and fine‐grain groups exhibited higher *r*‐value in Pearson correlation analysis around the segmentation event (time point zero). In the Free‐segmentation group, a significant time window ranging from 117 to 167 ms (*r*‐value_max_ = .530, *r*‐value_min_ = .340) was identified following the segmentation event. On the other hand, in the fine‐grain group, a significant time window spanning from −97 to −30 ms (*r*‐value_max_ = .495, *r*‐value_min_ = .315) was observed before the segmentation event. In order to compare the correlational results between groups, the number of time points showing a significant correlation with the behavioral outcome was extracted for the time periods before and after the segmentation event. A mixed‐effects ANOVA revealed an interaction of group and time period (*F*(1,79) = 1245.0, *p* < .001, *η*_p_^2^ = .940), establishing that the fine‐grain group had more significantly correlating time points than the Free‐segmentation group before the segmentation event (*t*(79) = −61.7, *p* < .001, *d* = 13.721), whereas the Free‐segmentation group had more significantly correlating time points than the Fine‐grain group after the segmentation event (*t*(79) = 6.7, *p* < .001, *d* = 1.482).

## 4 DISCUSSION

The current study examined the neural principles underlying how people segregate continuous incoming information in a given situation into meaningful units under different metacontrol‐relevant instructions. In doing so, our study connects EST (Zacks et al., 2007) with the metacontrol framework of cognitive control (Goschke & Bolte, 2014; Hommel, 2015), which assumes that metacontrol styles affect the degree of exclusivity versus inclusivity of information processing and event representation (Hommel & Colzato, 2017b). We examined two groups of healthy participants watching a movie and being instructed to press the space key whenever they found some meaningful units. The fine‐grain group was asked to define these meaningful units as small as they could, whereas the free‐segmentation group received no specific instructions on how to perform the event segmentation.

The behavioral data revealed that the two groups differed in how they performed event segmentation: more segments were determined by participants in the fine‐grain group than in the free‐segmentation group, replicating earlier observations (Kurby & Zacks, 2011). Moreover, the probability to set an event boundary given incoming information (changes in scenes in the movie) was higher in the fine‐grain group than in the Free‐segmentation group (Figure 2 and Table 1). Thus, as expected, the metacontrol‐relevant instruction changed the mode how incoming information was handled: participants with a fine‐grain instruction were more focused and restrictive in deciding on whether incoming information still fit to the currently active working event model. Cognitive control modulates whether coarser or finer grained segments of natural scenes may be formed.

The neurophysiological data provide more insights into the associated neural mechanisms. The across‐time MVPA (Figure 4, upper panels) revealed a similar pattern in both groups: Decoding was possible about 400 ms before an event boundary was set, which was related to modulations in fronto‐polar cortex activity (BA10) (i.e., higher activity in intervals containing a boundary than intervals without an event boundary). Importantly, it was previously shown that motor preparation processes only start approximately 135 ms before the response (Prochnow et al., 2024), so that it is unlikely that the decoding seen in the MVPA about 400 ms before the response reflects differences in motor activity. Moreover, the localization of modulations in the fronto‐polar cortex which is not associated with motor activity underlines that the difference between the conditions is not related to differences in motor activity. Likely, the fronto‐polar cortex performs an online monitoring of the relevance of behavioral options (Koechlin & Summerfield, 2007; Mansouri et al., 2017), enabling the concurrent tracking and evaluation of multiple hypotheses regarding potential future behavioral options (Mansouri et al., 2017). This shows similarities to predictive coding mechanisms (Pezzulo et al., 2024). This process is necessary during event segmentation where incoming information is continuously compared to information from the working event model and event schemata (Richmond & Zacks, 2017; Zacks, 2019) in order to partition information into encapsulated perception‐action episodes needed to inform subsequent goal‐directed behavior (Beste et al., 2023; Frings et al., 2020; Hommel, 2009). Such episodic information is likely handled downstream of the processes of the fronto‐polar cortex (Koechlin & Summerfield, 2007). It is therefore reasonable that a process (i.e., event segmentation) managing representational dynamics ultimately leading to such episodes is associated with cortical structures that are at the top of the processing hierarchy, and managed in midline cortical structures known to be important for self‐referential processing (Qin & Northoff, 2011). Intriguingly, recent findings suggest that the fronto‐polar cortex plays an essential role during so‐called working memory gating (Yu et al., 2022), which regulates whether or not the content of working memory representations is updated (Konjusha et al., 2023; Rac‐Lubashevsky & Kessler, 2016, 2018; Rempel et al., 2021). During event segmentation it is important to determine when the working event model as current representation needs to be updated. Therefore, the current findings provide a bridge between the concepts of event segmentation and working memory gating and, moreover, outline an important role of fronto‐polar cortex activity in event segmentation. The latter corroborates a possibly overarching role of fronto‐polar regions. The current findings suggest that a process shared by event segmentation and working memory gating might be to an online monitoring of the relevance of different behavioral options facilitating the pursuit and comparison of possible outcomes of upcoming behavioral options simultaneously.

After an event boundary was set, activity in fronto‐polar regions was modulated as well (Figure 4, lower panels). This is reasonable because once an event boundary has been set, the representational dynamics discussed above has to start a new (Richmond & Zacks, 2017; Zacks, 2019). Intriguingly, after an event boundary has been set, additional insular activity was evident in the fine‐grain group. The insula (BA13) serves a diverse range of cognitive functions, encompassing sensory processing, affective processing, and higher‐level cognition. Its substantial white matter connections with other brain regions underscore its participation in a broad spectrum of cognitive functions. In particular, the insular cortex likely reflects a hub‐region receiving and broadcasting information (Cauda et al., 2012; Droutman et al., 2015; Gogolla, 2017). Similar to fronto‐polar regions, the insular cortex is also involved in the monitoring of goal‐directed actions (Gogolla, 2017). In the fine‐grain group, these monitoring processes are intensified (see Section 3.1) because the metacontrol instruction emphasized fine‐grained event segmentation. Likely, the additional involvement of the insular cortex (BA13) in this group reflects the required increased monitoring of upcoming event, which also explains why there was also a concomitant modulation of fronto‐polar cortex activity. Of note, the stronger monitoring of the situation and stricter judging of incoming information to form event segments does not alter whether the previously formed event segment affects the newly opened event segment. This is shown in the temporal generalization results (Figure 3, lower panels), which demonstrate no group difference. There was off‐diagonal activity showing that representations from about 800 to 100 ms before the event boundary are reactivated between 300 and 800 ms after the event boundary, suggesting that previous representational content is reactivated (King & Dehaene, 2014). Since this was the case for both groups irrespective of the instruction, metacontrol does not affect whether representational dynamics from previous segments affect representational dynamics thereafter. In other words, the dynamics treat units of different sizes alike. This is an important outcome since the present form of the EST assumes that there is a complete shielding of information between adjacent event segments (Radvansky & Zacks, 2017), with no opportunity of re‐iterant processing of previous representational content. This is an unrealistic assumption in the first place, because understanding the meaning of one element of a complex event often depends on other preceding elements. Additionally, the efficiency of forming a current event representation has been frequently found to depend on the feature‐overlap with previous event representations (Hommel, 2004). On a related note, Wahlheim and Zacks (2019) proposed the Event Memory Retrieval and Comparison (EMRC) theory. According to this, current event features trigger the retrieval of recent, related event representations. Our present observations corroborate these theoretical assumptions on a neural level and show that representing an event is modulated by representations of preceding events. Given the similarities to working memory gating on a neural level (Konjusha et al., 2023; Rac‐Lubashevsky & Kessler, 2016, 2018; Rempel et al., 2021), it is possible that the removal of an event model from working memory is incomplete while a new event model is created. Alternatively, it is possible that immediately preceding event representations are not deleted entirely but are transferred to long‐term memory.

Importantly, the correlational analyses between the MVPA classification results (associated with fronto‐polar cortex activity) and the behavioral data revealed distinct patterns (i.e., group difference) for the free‐segmentation and the fine‐grain group (Figure 5). Before setting an event boundary, there were positive linear correlations between the MVPA classification and the likelihood to set an event boundary given a situational change. This indicates that people with higher distinctiveness of the representational neural patterns between BI and NBI were more sensitive to situational changes and set more event boundaries. This is in line with the conceptual assumptions of EST, according to which it is the degree of a mismatch between incoming information and the working event model that causes an event boundary to be set (Richmond & Zacks, 2017; Zacks, 2019). Importantly, such a positive correlation occurred more often (and with strong effect size) in the group asked to perform a fine‐grained segmentation. Thus, the metacontrol of event segmentation changes how likely it is that changes in the representation of events, and thus differences between incoming information and the working event model, will be translated into behavior. This process ultimately relates to differences in how restrictive incoming information is handled and how encapsulated episodes are formed to organize incoming information.

## 5 CONCLUSION

In summary, the study shows how metacontrol affects the mode with which people structure incoming information into encapsulated episodes. The fine‐grain group showed more segments and a higher likelihood to set event boundaries upon scene changes, which supports the notion that cognitive control influences segmentation granularity. On the neural level, representational dynamics was decodable 400 ms before the decision to close a segment and open a new one, and especially fronto‐polar regions are associated with this representational dynamic. Groups differed in their use of this representational dynamics to guide behavior and there was a higher sensitivity to incoming information in the fine‐grain group. Moreover, a higher likelihood to set event boundaries was reflected by activity increases in the insular cortex suggesting an increased monitoring of potentially relevant upcoming events. The study connects the EST framework with the meta‐control framework and relates these to overarching neural concepts of prefrontal cortex function.

## AUTHOR CONTRIBUTIONS

All authors had full access to the data, gave final approval for publication and agree to be held accountable for the work performed therein. Conceptualization: **B. H.**, **V. R.**, **C. B.**; Software: **X. Z.**, **A. P.** Investigation: **X. Z.**, **F. G.**, **A. P.**; Formal Analysis: **X. Z.**, **A. P.**; Writing – Original Draft: **X. Z.**, **A. P.**, **C. B.**; Writing – Reviewing & Editing: all authors; Visualization: **X. Z.**, **F. G.**, **A. P.**; Supervision: **C. B.**; Funding Acquisition: **B. H.**, **V. R.**, **C. B.**

## FUNDING INFORMATION

This work was supported by a grant from the Else‐Kröner Fresenius Stiftung (Key project) to C. B., B. H., and V. R. (2020_EKSE.105).

## CONFLICT OF INTEREST STATEMENT

The authors declare no conflict of interest.

## Data Availability

The data that support the findings of this study are openly available in OSF at https://osf.io/78xyk/?view_only=6d155924939f437c86a376f805e51256.
